# Unraveling the quality of implementation of sexual and reproductive health projects among adolescents and youths in low-income regions: a case study of the JADES 2 project in Niger in 2021

**DOI:** 10.11604/pamj.2024.47.123.42725

**Published:** 2024-03-19

**Authors:** Alphonse Euphrème Ahlou, Hemaho Beaugard Taboe, Canisius Fantodji, Jacques Saizonou

**Affiliations:** 1 Département Santé, Université Senghor à Alexandrie, Alexandrie, Egypte,; 2 Unité Départementale des Sciences de la Gestion, Université de Québec à Rimouski, Rimouski, Canada,; 3 Department of Mathematics, University of Florida, Gainesville, FL 32611, USA,; 4 Laboratoire de Biomathématiques et d´Estimations Forestières, Université d’Abomey-Calavi, Cotonou, Benin,; 5 Epidemiology and Biostatistics Unit, Centre Armand-Frappier Santé Biotechnologie, Institut National de la Recherche Scientifique (INRS), Université du Québec, 531 Boul des Prairies, Laval, Québec, Canada,; 6 Research Centre, Sainte-Justine University Hospital Centre (*CHU Sainte-Justine*), Montréal, Qébec, Canada,; 7 Institut Régional de Santé Publique Comlan Alfred Quenum (IRS/CAQ), Université d'Abomey-Calavi (UAC), Abomey-Calavi, Bénin

**Keywords:** Service quality, sexual, reproductive health, adolescent, youth, JADES 2 project, Niger

## Abstract

**Introduction:**

many young people globally grapple with early pregnancies and sexually transmitted diseases (STD), especially in regions like Africa and particularly Niger due to high poverty rates. Various sexual and reproductive health (SRH) projects aim to address these challenges. This study evaluated the JADES 2 project's implementation of SRH services provided focusing on the quality of these services in Niger in 2021.

**Methods:**

a cross-sectional evaluative study was conducted based on Donabedian and Bruce's theory from March 10 to August 15, 2021, in Niger. The evaluation was carried out through the analysis of supervision data, administration of questionnaires, and semi-structured interviews in 9 Integrated Health Centers and Youth-Friendly Centers. Across these sites, 203 adolescents and young people, as well as 9 healthcare workers involved in providing SRH services, were interviewed. The composite indicator in the field developed by the World Health Organization (WHO) in 2000 was used.

**Results:**

the quality score estimated for the project was 67% indicating that the services provided was of good quality. The majority (56%) of surveyed people were very satisfied with the services received, and 65% were aware of at least two methods of preventing Sexually transmitted infections (STIs) and three methods of preventing early pregnancies.

**Conclusion:**

the SRH services implemented are of acceptable quality. The study identified gaps in the process of service provision, particularly regarding confidentiality and the availability of inputs and medications.

## Introduction

The sexual and reproductive health (SRH) of adolescents and young people is a significant health and development challenge worldwide. Indeed, adolescents and young people constitute a valuable resource for countries; however, they face an increased risk of mortality and morbidity due to events that can hinder their physical, mental, and social well-being [1-4]. They have limited access to SRH advice and care, fearing identification, judgment, or discrimination [5]. This difficulty in accessing SRH services by adolescents and young people can also be attributed to the poor quality of services they receive in health centers. The quality of SRH services, though challenging to measure, is crucial in improving the performance of projects and programs for the sexual and reproductive health of adolescents and young people [4,6]. A study conducted in the Philippines highlighted the importance of the quality of family planning services. Indeed, when women received quality services, their utilization significantly increased [7]. It was observed that providing counseling to clients during family planning services would improve both short-term outcomes, such as knowledge enhancement and satisfaction with services, and long-term outcomes, such as increased birth spacing and continued use of modern contraceptive methods [8,9]. Furthermore, how adolescents and young people act based on the information and services received depends on their reception by the healthcare provider and the overall experience of their clinical visit. Also, their perceptions of the quality and satisfaction with care influence their commitment to continue services [10-12].

As defined by Donabedian 1988, a pioneer in healthcare quality assessment, there are three dimensions for achieving optimal quality of care: the structure (staffing, training, and qualifications of personnel, supplies, equipment, facilities, technology, and others.), the process (technical and interpersonal aspects, performance, and service compliance with good professional practices), and the outcomes (customer knowledge and satisfaction, health status, and other) [13]. According to Berwick, quality care should be effective, efficient, accessible, acceptable/patient-centered, equitable, and safe [14]. The WHO has developed global standards for healthcare for adolescents and young people based on the assessment of their needs [15]. There are eight standards, taking into account the following aspects: adolescents' health literacy, community support, appropriate service package, provider skills, facility characteristics, equity and non-discrimination, data and quality improvement, and adolescent participation [16,17]. Existing literature focuses on evaluating factors that affect the utilization of services, particularly clinical services, among adult women or couples. It emphasizes obtaining care without measuring satisfaction [18]. Studies specifically addressing adolescents and young people that integrate the quality of services as essential factors for the continued use of SRH services are nonexistent [13].

In Niger, data regarding the quality of SRH services for adolescents and young people are nonexistent. The JADES project (Jeunes Adolescents En Santé), an initiative led by Solthis, Equipop, and the NGO Lafia Matassa, was launched in 2017 and is currently in Phase II. Its general objective is to improve young people's access to Sexual and Reproductive Health Rights (SRHR) by working on strengthening both supply and demand. It also aims to enhance their capacity to reduce new human immunodeficiency virus (HIV) infections and unwanted pregnancies in Niger. Furthermore, it aims to increase the quality of SRH services offered to adolescents and young people. To achieve this mission, it is important to identify the factors, including the health center and services offered, provider and client characteristics, that are associated with the quality of sexual and reproductive health services for adolescents and young people aged 10 to 24 in Niger. Thus, this study intends to investigate the quality of sexual and reproductive health (SRH) services offered to adolescents and young people in Youth-Friendly Centers (YFC) and Integrated Health Centers (IHC) targeted by the JADES 2 project in Niger in 2021. Specifically, we aim to: 1) determine the level of quality of SRH services received by adolescents and young people in these centers, 2) ) assess the knowledge of SRH, perceptions, and satisfaction of adolescents and young people regarding the SRH services received.

## Methods

**Study design:** a cross-sectional study was conducted in Niger over a period of 6 months (March to August 2021) to evaluate the quality of SRH services offered by the JADES 2 project.

**Study setting and population:** Niger is a sub-Saharan African country with 51.6% of its population under the age of 15 [19]. According to the latest census (RGPH, 2012), 77% of young girls are married before the age of 18, with the average age of first marriage being 15.7 years [20]. The onset of first sexual intercourse occurs at 15.9 years. The adolescent fertility rate is the highest in the world (206‰ according to EDSN MICS IV in 2012), with a birth rate of 151/1000. This high fertility is accompanied by a significant maternal mortality rate: 34.4% of deaths recorded in public health service maternity wards occur in adolescents aged 15-19 due to obstetric complications. Unmet needs for SRH among adolescents and young people are high, with significant inequalities, especially concerning women in union. The HIV prevalence is 1% compared to 0.7% in the general population, the contraception rate among women of reproductive age is 13.1%, and there is limited access to family planning information at 16% [21].

The primary population of this study was the group of adolescents and young people aged 15 to 24 living in the intervention areas of the JADES 2 project, specifically in Niamey and Maradi. This area included seven Integrated Health Centers (IHC): Koira Kano Nord IHC, Recasement IHC, Gamkalé IHC, Aéroport I IHC; and Andoumé IHC, Zaria II IHC, Place du Chef IHC, and two youth-friendly Centers targeted by the project: National Center for Reproductive Health (NCRH), and National Reference Center for Youth (NRCY) Boukoki in Niamey. A non-probabilistic sampling technique was used to recruit adolescents and young. A comprehensive census was conducted for all adolescents and young people seen in consultation at the IHC and YFC targeted by the project during 4 weeks from June 7 to July 5, 2021. Our inclusion criteria were aged 15-24, visiting one of the centers during the survey period, and having not yet participated in the survey in a center. In addition to the population of adolescents and young on which this study focuses, we also recruited all (09) healthcare providers who were in service at the health centers listed above. Those who were absent during the survey period were excluded.

**Variables:** variables included health center, indicators of structure, and indicators of the process as defined in a theoretical model of Donabedian and Bruce (see below). The last two subgroups of variables determine the quality (good, medium, or low) of the SRH service provided. In addition, dependent variables regarding the adolescents and young included: knowledge of SRH, perception of services received, and satisfaction regarding the services received, and independent variables included health center, gender, age, education, marital status, affordability of the service, being at first visit in the center, have been explained how to avoid illness, and kindness of the healthcare workers.

### Data resource and measurement

**Data collection tool:** regarding the health center, a document grid was carefully prepared to collect supervision data. This tool was inspired by two dimensions of the theoretical model of Donabedian and Bruce: i) indicators of structure (general organization of the service, opening hours and waiting times in the centers, accessibility, hygiene, availability of equipment and inputs, training of personnel in SRH, health information system, referral system), and ii) indicators of process (welcome, health education, information system, respect for informed choice, confidentiality, non-discrimination, technical skills (consultation), adherence to protocols and national recommendations, involvement of young people and adolescents, community strategy). This grid considered the standards recommended by the WHO for the quality of services for adolescents and young people, on the one hand, and, on the other hand, the strategic plan for the health of adolescents and young people in Niger. Thus, it explored thirteen sub-dimensions of the quality of services for adolescents and young people using 71 binary questions (yes, no). A questionnaire was developed for adolescents and young people aged 15 to 24. The questions were extracted from various questions in the WHO standard on quality health services for adolescents and young people. They were updated and adapted to sexual and reproductive health in the Nigerians context, focusing on the third dimension of service quality. In addition to the variables listed above, we included sociodemographic variables (gender, age, education, marital status), affordability of the service, being at first visit in the center, have been explained how to avoid illness, and kindness of the reception. The questionnaire consisted of eighteen (18) questions (binary and multiple-choice). Pilot surveys were conducted to test the tool on 10 young people aged 15 to 24 at IHC Yantalla (not covered by the project). The same questionnaire was also tested with students from the Department of Environment and Health at the University of Senghor in Alexandria, Egypt. These tests helped refine the questionnaire.

**Data collection:** to minimize the error that occur during data collection, the questionnaire and the documentary grid were digitized using Kobocollect platform [22] and then installed on smartphones that were used to collect the data. The questionnaire was administered by 09 trained investigators between June 7 and July 5, 2021. The supervision data were extracted from the supervision databases in March 2021. A semi-direct interview was conducted in person by the principal investigator in the 9 centers to document the socio-professional characteristics of the providers and their perceptions on the quality of sexual health services for adolescents and young people. Specifically, data collected covered: provider training in SRH, preferences of adolescents and young people regarding offered SRH services, expectations of young people in terms of SRH, current perceptions of the quality of services offered to adolescents and young people, assessment of the organization of the quality improvement system, obstacles to quality, and solution approaches.

**Sample size:** two hundred and three adolescents and young with aged between 15 and 24 who are beneficiaries of JADES 2 project in the different health centers are considered as our sample size obtained by the method explained above. The supervision data were collected for all the health centers targeted by the project and 9 healthcare providers were interviewed.

**Data analysis:** the supervision data obtained were thoughtfully processed using the Statistical software STATA version 13 combined with Microsoft Excel. In order to evaluate the overall internal consistency of the items used to determine the quality of the service provided, we used Cronbach's alpha [23]. To determine the level of quality of the centers covered by the project, a scoring system was assigned to various indicators of the structure and process of SRH service quality. Here, a 'Yes' counted as one point, and a 'No' counted as zero. The sum provided the overall score of the centers as a percentage in relation to the structure and process indicators. The United Nations Population Fund (UNFPA) approach, commonly used in developing countries, was employed as a reference to classify health facilities based on their quality [24]. Therefore, the quality score was categorized into 3 classes: 1) low quality if the score is less than 50%, 2) medium quality if the score is between 50-74%, and 3) good quality if the score is above 75%. Regarding knowledge in SRH, perceptions, and satisfaction of adolescents and young people, the analysis was done by the health center. We first conduct descriptive analysis, including comparisons of frequencies with Chi-square or Fisher exact test for qualitative variables and central tendency measure (average) for quantitative variables. Logistic regression analysis was performed in STATA, and Odds ratios (OR) were estimated with their 95% confidence intervals.

**Ethical consideration:** we received approval from the Ministry of Health of Niger to conduct this study. All the data were collected from voluntary, informed, and consent participants. No personal data were collected, and the confidentiality of the responses was ensured by the digitalization of the data collection tools. The database was saved on a laptop with a strong security password.

## Results

**Figure 1:**
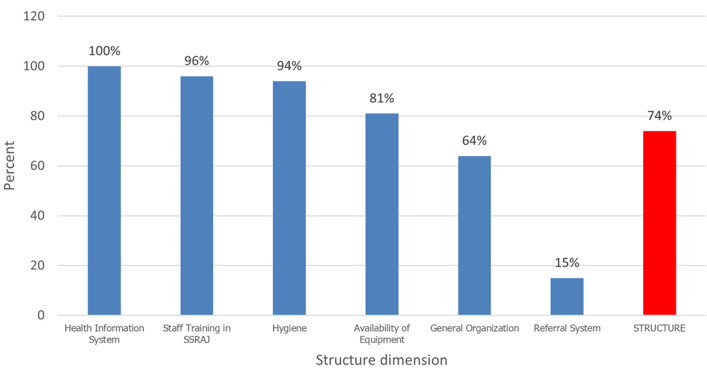
quality score based on structural indicators in Integrated Health Centers and Youth-Friendly Centers targeted by the JADES project in 2021

**Figure 2:**
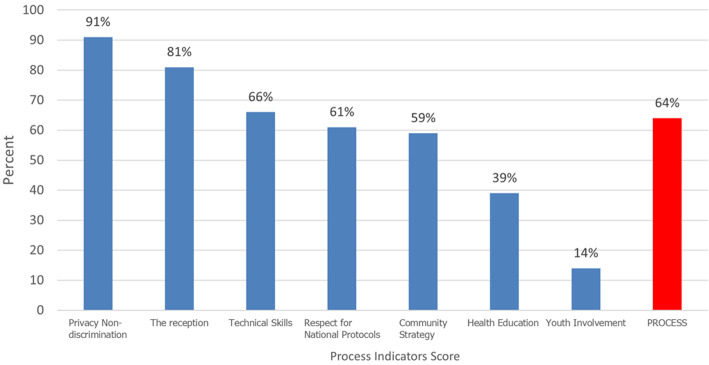
quality score based on process indicators in Integrated Health Centers and Youth-Friendly Centers targeted by the JADES project in 2021

A total of 71 questions (exploring the structure and process of the theoretical model of Donabedian and Bruce) with responses (yes/no) were used to determine the quality score of the implementation of sexual and reproductive health services for adolescents and young people in the IHC and YFC targeted by the JADES 2 project. The overall internal consistency of these questions (Cronbach's alpha) was 0.93 meaning that the indicators used to assess the quality of SRH services correctly explored (93%) all the dimensions evaluated. The cumulative scores of the quality structure indicators were 74% in the IHC and YFC. Performances of around 100% were achieved for indicators of the health information system, 94% for hygiene, and 96% for personnel training in SRHR. However, the referral system (15%) appeared to be the weak point in the structure of indicators of service quality in the IHC and YFC targeted by the project (Figure 1). For the cumulative scores of process indicators, they were 64% in the IHC and YFC. Performances of around 100% were achieved for indicators of the health information system, 94% for hygiene, and 96% for personnel training in SRHR in the centers. However, youth involvement (14%) and health education (39%) appeared to be the weak points in the process indicators of service quality in the IHC and YFC targeted by the project (Figure 2). The overall score of SRH services implemented in the IHC and YFC targeted by the project was around 67%. Our result revealed that the structure score (74%) was higher than the process score (64%). The IHC and YFC targeted by the JADES 2 project provide a service of moderate quality to adolescents and young people in terms of sexual and reproductive health, with disparities between the service delivery structures targeted by the project.

**Knowledge in SRH, satisfaction, and perceptions:** the average age of the adolescents and young participants in this study is 21 years, and the majority (86%) were females. The predominant religion was Islam (97%), and 7 out of 10 of them had a spouse. Regarding education, 35% of participants were not enrolled in school. Most adolescents and young people (73%) had visited the project-targeted centers at least once, while 26% were visiting for the first time. More than half of the participants (56%) were very satisfied with the SRH services received in the IHC and YFC targeted by the JADES project. Only 2%, 7%, and 34% of participants were respectively dissatisfied, slightly satisfied, and rather satisfied. Adolescents and young people who frequented CNRJ-B (OR=1.66; 95% CI: 0.43-2.90), IHC Andoumé (OR=2.03; 95% CI: 0.56-3.51) and IHC Recasement (OR=1.56; 95% CI: 0.32-2.80) were more likely to know three methods of preventing early and unwanted pregnancies. Also, knowing methods to prevent STIs/HIV significantly (p < 0.001) increased knowledge of methods to prevent early or unwanted pregnancies. Adolescents and young people who knew how to prevent STIs/HIV were 19 times more likely to know three methods of preventing pregnancies (OR=19.88; 95% CI: 9.48-41.67). However, adolescents who visited IHC Aéroport (OR=0.07; 95% CI: 0.02-1.23), and IHC Zaria 2 (OR=0.30; 95% CI: 0.13-0.72) were unlikely to know three methods of preventing early or unwanted pregnancies (Table 1).

**Table 1:** logistic analysis of knowledge of means of preventing early and unwanted pregnancies with individual characteristics, Integrated Health Centers and Youth-Friendly Centers, and perception of services received by adolescents and youth (n=203)

Knowledge of methods ^a^ for preventing early and unwanted pregnancies						
Variables	Yes	%	No	%	OR (95% CI)	P-value
**Boukoki NRCY**						
No	107	61.14	68	38.86	1 (reference)	
Yes	25	89.29	3	10.71	5.26 (1.53, 18.17)	0.008
**NCRH**						
No	132	67.35	64	32.65		
Yes	0	0.00	7	100.00	NA	>0.9
**Aeroport IHC**						
No	128	72.32	49	27.68	1 (reference)	
Yes	4	15.38	22	84.62	0.07 (0.02, 1.23)	< 0.001
**Gamkale IHC**						
No	115	65.34	61	34.66	1 (reference)	
Yes	17	62.96	10	37.04	1.47 (0.94, 2.07)	0.809
**Andoume IHC**						
No	108	61.02	69	38.98	1 (reference)	
Yes	24	92.31	2	7.69	7.61 (1.75, 33.44)	0.007
**Koara Kano IHC**						
No	141	68.78	64	31.22	1 (reference)	
Yes	18	72.00	7	28.00	3.12(0.67, 14.43)	0.145
**Place du Chef IHC**						
No	18	72.00	7	28.00	1 (reference)	
Yes	114	64.04	64	35.96	3.09 (0.57, 3.63)	0.437
**Recasement IHC**						
No	109	61.58	68	38.42	1 (reference)	
Yes	23	88.46	3	11.54	4.75 (1.37, 16.44)	0.013
**Zaria 2 IHC**						
No	122	68.54	56	31.46	1 (reference)	
Yes	10	40.00	15	60.00	0.30 (0.13, 0.72)	0.007
**Gender**						
Female	22	78.57	6	21.43	1 (reference)	
Male	110	83.33	22	16.67	2.15 (0.83, 5.58)	0.112
**Age**						
15-19 year old	105	66.46	53	33.54	1.30 (0.67, 2.58)	0.424
20-24 year old	27	60.00	18	40.00	1 (reference)	
**Education level**						
Not enrolled	13	81.25	3	18.75	1 (reference)	
Enrolled^b^	132	66.00	68	34.00	0.40 (0.11, 1.46)	0.168
**First visit in IHC or in YFC**						
No	102	68.46	47	31.54	1 (reference)	
Yes	30	55.56	24	44.44	0.57 (0.2, 1.08)	0.09
**Inquire about sexual relationships**						
No	63	66.32	32	33.68	1 (reference)	
Yes	69	63.89	39	36.11	0.90 (0.50, 1.59)	0.717
**Explain how to avoid illnesses**						
No	21	60.00	14	40.00	1 (reference)	
Yes	111	66.07	57	33.93	1.29 (0.61, 2.71)	0.494
**Kind, caring, and make you feel comfortable**						
No	13	86.67	2	13.33	1 (reference)	
Yes	119	63.30	69	36.70	0.26 (0.05, 1.20)	0.087
**Satisfaction**						
No	12	63.16	7	36.84	1 (reference)	
Yes	120	65.22	64	34.78	1.08 (0.41, 2.91)	0.858
**Knowledge of at least 2 methods of preventing STIs**						
No	17	24.29	53	75.71	1 (reference)	
Yes	115	86.47	18	13.53	19.88 (9.48, 41.67)	< 0.001

a : ‘yes’ if know at least three methods and ‘no’ else; ^b^: enrolled if the education level is at lest the primary level; NRCY: National Reference Center for Youth; NCRH: National Center for Reproductive Health; IHC: Integrated Health Center; STIs: sexually transmitted infections

Furthermore, adolescents and young people who visited CNRJ (OR=17.46; 95% IC: 2.31-131.63), IHC Andoumé (OR=7.46; 95 CI: 1.69-32.45), IHC recasement (OR=4.66; 95 CI: 1.34-16.11), were male (OR=5.15; 95% CI: 1.49-17.63), and knew three methods of preventing pregnancies (OR=19.88; 95% CI: 9.48-41.67) were likely to know two methods of preventing STIs/HIV. However, adolescents who visited IHC Aéroport (OR=0.09; 95% CI: 0.03-0.25) were at a higher risk of not knowing two methods of preventing STIs/HIV (Table 2). Adolescents and young people whose waiting time was short (OR=1.53; 95% CI: 0.46-2.95), those whose choices were respected (OR=32.45; 95% CI: 5.75-181.27), those who were confident (OR=10.07; 95% CI: 3.25-31.18), treated in a friendly manner (OR=7.45, 95% CI: 1.85-20.69), and those who found services cheaper (OR=7.53; 95% CI: 2.38-64.07) were more likely to be satisfied with the received SRH services. In contrast, visiting IHC Koara Kano (OR=0.03; 95% CI: 0.01-0.13) would decrease the chance of being satisfied (Table 3).

**Table 2:** logistic analysis of knowledge of means of preventing STIs/HIV with individual characteristics, Integrated Health Centers and Youth-Friendly Centers, and perception of services received by adolescents and youth (n=203

Knowledge of means of preventing STIs/HIV						
Variables	Yes^a^	%	No	%	OR (95% CI)	P-value
**Boukoki NRCY**						
No	106	60,57	69	39,43	1 (reference)	
Yes	27	96.43	1	3.57	17.46 (2.31;131.63)	0.005
NCRH						
No	133	67.86	63	32.14	1 (reference)	
Yes	0	0.00	7	100.00	-	>0.9
**Aeroport IHC**						
No	128	72.32	49	27.68	1 (reference)	
Yes	5	19.23	21	80.77	0.09 (0.03; 0.25)	< 0.001
**Gamkale IHC**						
No	117	66.48	59	33.52	1 (reference)	
Yes	16	59.26	11	40.74	0.74 (0.32; 1.66)	0.464
**Andoume IHC**						
No	109	61.58	68	38.42	1 (reference)	
Yes	24	92.31	2	7.69	7.46 (1.69; 32.45)	0.007
**Koara Kano IHC**						
No	125	65.79	65	34.21	1 (reference)	
Yes	8	61.54	5	38.46	0.16 (0.26; 2.63)	0.755
**Place du Chef IHC**						
No	120	67.42	58	32.58	1 (reference)	
Yes	13	52.00	12	48.00	0.52 (0.22; 1.20)	0.133
**Recasement IHC**						
No	110	62.15	67	37.85	1 (reference)	
Yes	23	88.46	3	11.54	4.66 (1.34; 16.11)	0.015
**Zaria 2 IHC**						
No	116	65.17	62	34.83	1 (reference)	
Yes	17	68.00	8	32.00	1.12 (0.46; 2.77)	0.78
**Gender**						
Female	108	61.71	67	38.29	1 (reference)	
Male	25	89.29	3	10.71	5.15 (1.49; 17.63)	0.009
**Age**						
15 -19 year old	106	67.09	52	32.91	1.34 (0.69; 2.66)	0.378
20 -24 year old	27	60.00	18	40.00	1 (reference)	
**Education level**						
Not enrolled	14	87.50	2	12.50	1 (reference)	
Enrolled	68	36.36	119	63.64	0.25 (0.05; 1.12)	0.072
**First visit in IHC or in YFC**						
Yes	34	62.96	20	37.04	1 (reference)	
No	99	66.44	50	33.56	0.86 (0.80; 1.63)	0.645
**Inquire about sexual relationships**						
No	62	65.26	33	34.74	1 (reference)	
Yes	71	65.74	37	34.26	1.02 (0.57; 1.82)	0.943
**Explain how to avoid illnesses**						
No	20	57.14	15	42.86	1 (reference)	
Yes	113	67.26	55	32.74	1.53 (0.72; 3.22)	0.254
**Kind, caring, and make you feel comfortable**						
No	11	73.33	4	26.67	1 (reference)	
Yes	12	15.38	66	84.62	0.33 (0.20; 2.18)	0.51
**Satisfaction**						
No	9	47.37	10	52.63	1 (reference)	
Yes	124	67.39	60	32.61	2.29 (0.88; 5.92)	0.087
Knowledge of at least 2 methods of preventing STIs						
No	18	25.35	53	74.65	1 (reference)	
Yes	115	87.12	17	12.88	19.88 (9.48; 41.67)	< 0.001

NRCY: National Reference Center for Youth; NCRH: National Center for Reproductive Health; IHC: Integrated Health Center; STIs: sexually transmitted infections, a=Yes if Know at least two means of preventing STIs/HIV

**Table 3:** logistic analysis of satisfaction of adolescents and youth with services received on individual characteristics, Integrated Health Centers and Youth-Friendly Centers, and perception of services received (n=203).

Satisfaction^a^						
Variables	Yes	%	No	%	OR (95% CI)	P-value
**Boukoki NRCY**						
No	157	89.71	18	10.29	1 (reference)	
Yes	27	96.43	1	3.57	3.06 (0.39; 24.04)	0.281
**NCRH**						
No	177	90.31	19	9.69	1 (reference)	
Yes	7	100.00	0	0.00	-	>0.9
**Aeroport IHC**						
No	158	89.27	19	10.73	1 (reference)	
Yes	26	100.00	0	0.00	-	>0.9
**Gamkale IHC**						
No	160	90.91	16	9.09	1 (reference)	
Yes	24	88.89	3	11.11	0.80 (0.21; 2.94)	0.738
**Andoume IHC**						
No	158	89.27	19	10.73		
Yes	26	100.00	0	0.00	-	>0.9
**Koara Kano IHC**						
No	178	94.18	11	5.82	1 (reference)	
Yes	5	38.46	8	61.54	0.03 (0.01; 0.13)	< 0.001
**Place du Chef IHC**						
No	160	89.89	18	10.11	1 (reference)	
Yes	24	96.00	1	4.00	2.69 (0.34; 21.11)	0.344
**Recasement IHC**						
No	161	90.96	16	9.04	1 (reference)	
Yes	23	88.46	3	11.54	0.76 (0.20; 2.80)	0.684
**Zaria 2 IHC**						
No	162	91.01	16	8.99	1 (reference)	
Yes	22	88.00	3	12.00	0.72 (0.19; 2.66)	0.63
**Gender**						
Male	26	92.86	2	7.14	1 (reference)	
Female	158	90.29	17	9.71	1.39(0.30; 6.35)	0.666
**Age**						
15-19 year old	144	91.14	14	8.86	1.28 (0.44; 3.78)	0.648
20- 24 year old	40	88.89	5	11.11	1 (reference)	
**Marital status**						
Single	126	89.36	15	10.64	0.58 (0.18; 1.82)	0.35
Married	58	93.55	4	6.45	1 (reference)	
**First visit to the IHC or YFC**						
Yes	49	90.74	5	9.26	1 (reference)	
No	135	90.60	14	9.40	1.03 (0.45; 2.94)	0.976
**Short waiting time^b^**						
No	69	83.13	14	16.87	1 (reference)	
Yes	114	95.80	5	4.20	4.61 (1.58; 19.10)	0.005
**Respect choice**						
No	2	28.57	5	71.43	1 (reference)	
Yes	182	92.86	14	7.14	32.45 (5.75; 181.27)	< 0.001
**Establishing trust**						
No	10	58.82	7	41.18	1 (reference)	
Yes	174	93.55	12	6.45	10.07 (3.25; 31.18)	< 0.001
**Kind, caring, and make you feel comfortable**						
No	10	66.67	5	33.33	1 (reference)	
Yes	174	92.55	14	7.45	6.17 (1.85; 20.69)	0.003
**No discrimination**						
No	181	90.95	18	9.05	1 (reference)	
Yes	3	75.00	1	25.00	0.30 (0.02; 3.00)	0.306
**Affordable (economic)**						
No	61	80.26	15	19.74	1 (reference)	
Yes	123	96.85	4	3.15	7.53 (2.38; 64.07)	0.001

a : satisfaction is ‘yes’ if the response is very satisfied or rather satisfied and ‘no’ if the response is dissatisfied/not satisfied; ^b^: if the respondent stated waiting for over 45 minutes; NRCY: National Reference Center for Youth; NCRH: National Center for Reproductive Health; IHC: Integrated Health Center

**Interviews with health professionals:** the interviews focused primarily on the services most demanded by young people and adolescents attending the centers, health workers' perceptions of the quality of services offered, their appreciation of the quality evaluation system, the challenges they face, and their suggestions to improve the quality of SRH services for adolescents and youth in Niger. Out of the 9 informants, all were women, two were general practitioners, two were nurses, four were midwives, and one was a senior technician in sexual and reproductive health. Their age ranged from 32 to 44 years, with an average age of 34 years. Eight out of 9 agents had received comprehensive training in SRH, and this training was less than 1 year. The majority had less than 2 years of experience in the various IHC and YFC targeted by the project. The collected statements revealed that lack of information, financial means, geographical accessibility, stigmatization, fear, and religion were obstacles to the attendance of adolescents and youths at the IHC and YFC targeted by the JADES 2 project: *"In my center, the problem is accessibility; young people do not have access to the center mainly due to the lack of information about the services provided at the IHC [...], the lack of money to honor prescriptions even if the consultation is free."*- Mrs. X, Midwife. *"The lack of information is the big problem [...], stigmatization, fear, and religion. Religion does not prevent them from coming, but with religion, as soon as you see a young person frequenting a health center, it means she is already corrupted [...] she will take condoms; it means she is pregnant. Religion says you should not engage in sexual activity before marriage. While the center is not just about sex or anything else, young people can come for information in general."*- Mrs. Y, Nurse.

**Demand for services and perceptions of health professionals:** the services most requested by adolescents and youth in the IHC and YFC targeted by the JADES 2 project were those related to the management of STIs, contraception, and HIV testing. *"Some come for family planning and disease management services, rarely for maternity services. Young boys often come to get condoms. For counseling, it's rare except during field consultation sessions."*- Mrs. X, Physician. *"They also come for STI management and contraception [...] especially at night, young women come to get condoms."*- Mrs. Z, Midwife. Most health workers were aware that young people turn to their center to find solutions to their problems, and both reception and confidentiality are crucial in this search for solutions. *"Their expectation is to have solutions to the problem that brings them. They come to be satisfied, to be taken care of as it should be [...] and especially without a prescription, free care. It's mainly about reception, confidentiality, and building trust."*- Mrs. X, Nurse. *"When a young person comes, it means there is a problem; they want their problem to be solved, whether it's a health problem or in life. Just last Saturday, someone came, he had a problem to explain to me, but when he saw that we were not alone, he refused to explain his problem."*- Mrs. Y, Physician.

Training of health workers had helped remove barriers to the care of adolescents and youth. It had prevented prejudices and separated personal values from professional values. *"As I am used to receiving 13-year-olds who come to get condoms, nothing surprises me in front of cases. A person who is sexually active is very likely to have an STI; when a young person comes, no matter the problem, my face always remains composed. I take care of them properly, trying to keep calm."*- Mrs. Y, Midwife. Confidentiality and the lack of inputs were the main obstacles to providing quality services in the IHC and YFC targeted by the JADES 2 project. *"For confidentiality, we can say yes and no. Yes, because young people can go behind. No, because when young people come, there may be people in the room [...] there is no dedicated space."*- Mrs. Y, Midwife. *"The problems are enormous, in terms of center attendance; young people do not have a special place for their care. Here, it is generally women who attend."*- Mrs. Y, Midwife.

## Discussion

We evaluated an overall quality score of 67% for the project. This score is higher than those reported by Mulugeta *et al*. [25] in Ethiopia (48%) in 2019 and comparable to the score reported in China (67%) by the project China Family planning association (CFPA) & PATH [26]. When evaluating structural and process dimensions of SSR service quality, it was observed that 4 out of 9 centers achieved a score higher than 75%. However, none of the IHC or YFC targeted by the project reached a score of 75% for process indicators. In general, services offered were of average quality, and none of the 9 centers scored below 50%. This suggests that the dimension hindering the quality of SSR services in the targeted centers lies in the interaction between service providers and adolescents and youth. These results generally surpass those reported in the literature. For instance, Mulugeta *et al*. (2019) found lower scores for both structural and process quality dimensions [25]. All IHC and YFC targeted by the JADES 2 project scored well in indicators related to the health information system (100%), hygiene (94%), and staff training in DSRHAY (Directorate of Sexual and Reproductive Health of Adolescents and Youths) (96%). This success is attributed to regular skills reinforcement in the targeted centers, including training in DSRHAY, gender-based violence (GBV), and sexual and reproductive health. However, deficiencies were noted in the referral system (15%), material availability (81%), and overall organization (66%). Notably, the lack of a specific space for adolescent and youth care in 6 out of 9 centers was highlighted, impacting the quality of services. While 91% of centers claimed to offer services with confidentiality, the actual practice raised concerns during interviews. The lack of a dedicated space for young individuals and the fear of encountering certain people in the service area posed confidentiality issues. This result is similar to the findings of Mulugeta *et al*. [25] and Munea *et al*. [2,4].

During interactions between providers and adolescents, providers seldom reminded clients of the confidential nature of the consultation, and services available were not communicated in 85.71% of cases. The use of national protocols for adolescent SRH was observed in 61% of centers, indicating room for improvement, but better than those reported by Thongmixay *et al*. [2,7]. The services most demanded by adolescents and youth were related to STI management, contraception, and HIV testing. Approximately 56% of participants expressed high satisfaction with SSR services, while 2% were dissatisfied. Factors influencing satisfaction included short waiting times, respect for choice, building trust, friendly reception, and economic accessibility of services. However, there was a significant difference in satisfaction between centers, suggesting that even with high-quality scores, the patient-provider interaction could impact satisfaction negatively. The JADES 2 project excels with high-quality supervisory data, providing detailed insights into Structural and Process dimensions of SRH service quality. Limitations involve a narrow focus on specific targets, hindering generalizability to the broader Nigerian context. Potential bias may stem from trust in sampled centers. The evaluation tool overlooks management dimensions and lacks detailed explanations for the minimum package of activities during adolescent and youth SRH care. Finally, the largest sample size could be useful to reinforce the robustness of our results.

## Conclusion

The quality of SRH services for adolescents and youths is crucial for the success of projects and programs in this area. The JADES´ project has made significant strides in improving the competence of providers and providing necessary equipment for quality care. However, challenges remain, especially in organizational aspects and the interaction between providers and adolescents. Addressing these challenges is essential for achieving the objectives of reducing early and unwanted pregnancies, as well as STIs and HIV. Offering high-quality services will be a valuable investment in the well-being of future generations, as well-educated parents in SRH will pass on their knowledge to their offspring.
